# 2-Heptyl-Formononetin Increases Cholesterol and Induces Hepatic Steatosis in Mice

**DOI:** 10.1155/2013/926942

**Published:** 2013-04-29

**Authors:** Charlotte Andersen, Janne G. Schjoldager, Christian G. Tortzen, Andreas Vegge, Majbritt R. Hufeldt, Mette T. Skaanild, Finn K. Vogensen, Karsten Kristiansen, Axel K. Hansen, John Nielsen

**Affiliations:** ^1^Department of Veterinary Disease Biology, Faculty of Health and Medical Sciences, University of Copenhagen, 1870 Frederiksberg C, Denmark; ^2^Department of Chemistry, Faculty of Science, University of Copenhagen, 1870 Frederiksberg C, Denmark; ^3^Department of Food Science, Faculty of Science, University of Copenhagen, 1870 Frederiksberg C, Denmark; ^4^Department of Biology, Faculty of Science, University of Copenhagen, 2200 Copenhagen N, Denmark; ^5^Department of Drug Design and Pharmacology, Faculty of Health and Medical Sciences, University of Copenhagen, 2100 Copenhagen Ø, Denmark

## Abstract

Consumption of isoflavones may prevent adiposity, hepatic steatosis, and dyslipidaemia. However, studies in the area are few and primarily with genistein. This study investigated the effects of formononetin and its synthetic analogue, 2-heptyl-formononetin (C7F), on lipid and cholesterol metabolism in C57BL/6J mice. The mice were fed a cholesterol-enriched diet for five weeks to induce hypercholesterolemia and were then fed either the cholesterol-enriched diet or the cholesterol-enriched diet-supplemented formononetin or C7F for three weeks. Body weight and composition, glucose homeostasis, and plasma lipids were compared. In another experiment, mice were fed the above diets for five weeks, and hepatic triglyceride accumulation and gene expression and histology of adipose tissue and liver were examined. Supplementation with C7F increased plasma HDL-cholesterol thereby increasing the plasma level of total cholesterol. Supplementation with formononetin did not affect plasma cholesterol but increased plasma triglycerides levels. Supplementation with formononetin and C7F induced hepatic steatosis. However, formononetin decreased markers of inflammation and liver injury. The development of hepatic steatosis was associated with deregulated expression of hepatic genes involved in lipid and lipoprotein metabolism. In conclusion, supplementation with formononetin and C7F to a cholesterol-enriched diet adversely affected lipid and lipoprotein metabolism in C57BL/6J mice.

## 1. Introduction

Over the last decade there has been a pronounced increase in the interest of the physiologic and pharmacologic effects of bioactive compounds. Of particular interest in relation to human health is a group of naturally occurring compounds called isoflavones which exhibit both hormonal and nonhormonal properties. These compounds are found in various legumes including soybean, green bean, and alfalfa sprout [1]. Originally isoflavones were studied for their effects on hormone-sensitive cancers, osteoporosis, menopause, and heart diseases [2]. However, recently the focus has been directed towards their effects on lipid metabolism. Data obtained from animal experiments as well as clinical and epidemiological studies suggest that consumption of isoflavones may prevent obesity [3, 4], type 2 diabetes [4], atherosclerosis [5], nonalcoholic fatty liver disease [6], and dyslipidaemia [7, 8]. Thus isoflavones might be useful in targeting the metabolic syndrome.

The most frequently studied isoflavone is genistein followed by daidzein. Supplementation with high doses of genistein or daidzein to rodents fed high-fat diets substantially decreases body weight and fat mass [9–11], lowers the plasma levels of total cholesterol, triglyceride, and LDL-cholesterol [9, 10, 12, 13], and protects against the development of hepatic steatosis [9, 10, 14, 15]. Thus, supplementation with isoflavones or synthetic analogues could potentially be used in prevention and/or treatment of obesity, dyslipidaemia, and hepatic steatosis.

Formononetin is an *O*-methylated isoflavone present in different bean types at various levels [16]. Previously extracts containing formononetin have been found to have an influence on fat metabolism [17, 18]. However, as extract also contains a range of other compounds, it is difficult to make solid conclusions on the effects of formononetin based on these studies. One study also shows a cardioprotective effect of a derivative of formononetin [19], suggesting that formononetin and derivatives of formononetin could have a positive influence on obesity and related disorders. However, the effects of formononetin remain putative owing to the existence of a range of other compounds found in extracts. 

A newly synthesised analogue of formononetin, 2-heptyl formononetin (C7F), affected lipid accumulation, lipolysis, and peroxisome proliferator-activated receptor *γ* (PPAR*γ*) activation *in vitro* more potently than synthetic formononetin (manuscript in preparation). Therefore, we aimed to investigate if formononetin and C7F positively affected lipid and cholesterol metabolism in C57BL/6 mice fed a cholesterol-enriched diet by assessing the effect of formononetin and C7F on body weight and composition, glucose tolerance, plasma lipid composition, hepatic steatosis, and expression of genes involved in lipid metabolism and phase I and II metabolism. 

## 2. Materials and Methods

### 2.1. Synthesis of Compounds

#### 2.1.1. General Experimental Information

Commercially available reagents (Sigma-Aldrich, Germany) were used without further purification unless otherwise noted. Solvents used for the synthesis were of analytical grade, dried over activated 4Å molecular sieves when necessary (all solvents used under dry conditions had a water content of <25 ppm measured by coulometric Karl Fischer titration). Analytical thin layer chromatography was performed using precoated silica gel 60 F254 plates (Merck, Germany) and visualized using either UV light or potassium permanganate stain. 

Column chromatography was performed on Merck Kiselgel 60 (0.015–0.040 mm) using the dry column vacuum chromatography (DCVC) technique [20]. High-performance liquid chromatography was performed on a Waters 2525 system equipped with a Waters 2996 photodiode array detector and a Waters 2767 Sample Manager using a 100 mm × 19 mm i.d. XTerra prep MS C18 column (Waters Corp., MA, USA) with a gradient of acetonitrile in Milli-Q water with a flow of 15 mL/min (Waters). Melting points were measured on a Reichert melting point microscope, model N254-1R (Austria). ^1^H and ^13^C NMR spectroscopic data were recorded on a Bruker Avance 300 (Bruker BioSpin MRI, Germany) using deuterated solvents as a lock. Chemical shifts are reported in parts per million relative to the residual solvent peak (^1^H NMR) or the solvent peak (^13^C NMR) as the internal standard. Accurate mass determinations were performed on a Micromass LCT apparatus (UK) equipped with an AP-ESI probe calibrated with Leu-Enkephalin (556.2771 g/mol). All spectrophotometric measurements were performed on a Shimadzu UV-2101PC UV-vis scanning spectrophotometer with automatic cell changer and a temperature-controlled water-jacket-regulated cell holder (Shimadzu Corp., Japan).

#### 2.1.2. Synthesis of C7F

2,4-Dihydroxy-4′-methoxy-deoxybenzoin (2.5 g, 9.7 mmol) was dissolved in 50 mL of dry THF. The stirred solution was cooled to 0°C, and triethylamine (4.1 mL, 3 eq.) was added. After 5 min. octanoyl chloride (3.7 mL, 2.2 eq.) was added, and the cooling was stopped. After 15 min. the reaction mixture was acidified with 50 mL of 1 M HCl. The yellow solution was extracted twice with EtOAc, and the combined organic phases were washed with water, sat. NaHCO_3_ aq., and brine. After removal of the solvent, the resulting residue was dried *in vacuo*. Purification by dry column vacuum chromatography (EtOAc/Heptane on silica—5% gradient) yielded the diester (4.7 g, 95%).

^13^C NMR (75 MHz, CDCl_3_): *δ* = 197.03, 172.02, 171.45, 158.75, 153.98, 150.20, 130.98, 130.69, 126.17, 119.09, 117.50, 114.25, 55.37, 47.43, 34.50, 34.41, 31.78, 29.18, 29.15, 29.08, 29.02, 24.91, 24.65, 22.74, 14.20 ppm.

^1^H NMR (300 MHz, CDCl_3_) *δ* = 7.80 (d, *J* = 8.6 Hz, 1H), 7.26 (s, 1H), 7.12 (d, *J* = 8.7 Hz, 2H), 7.06 (dd, *J* = 8.6, 2.2 Hz, 1H), 6.94 (d, *J* = 2.2 Hz, 1H), 6.86 (d, *J* = 8.7 Hz, 2H), 4.11 (s, 2H), 3.79 (s, 3H), 3.82–3.75 (m, 4H), 1.80–1.62 (m, 4H), 1.47–1.18 (m, 16H), 0.94–0.79 (m, 6H) ppm.

2,4-Dihydroxy-4′-methoxy-deoxybenzoin (4.7 g, 9.2 mmol) was dissolved into 60 mL of anhydrous THF and DBU (1.6 mL, 1.1 eq.) was added. The reaction mixture was heated to reflux for 1 hour, at which point 5 mL conc. HCl was added and the heating was continued for 1 hour. The reaction was monitored by TLC, and after complete conversion NaOH (6 mL, 10 M) was added and the solution was refluxed for another 30 min. The reaction was again monitored by TLC until complete conversion was observed. At this point, 1 M HCl was added until the reaction mixture was neutralised. The yellow solution was cooled and extracted twice with EtOAc. The combined organic phases were washed with water, sat. NaHCO_3_ aq., and brine. After removal of solvent, the resulting residue was dried *in vacuo*. The residue was purified by dry column vacuum chromatography (EtOAc/heptane on silica 5% gradient) yielding C7F (2.63 g, 78%) as a white foam.

^13^C NMR (75 MHz, CDCl_3_) *δ* = 177.82, 168.02, 162.83, 159.37, 158.17, 131.72, 127.68, 125.02, 122.37, 115.73, 114.13, 102.77, 77.58, 77.16, 76.74, 55.36, 32.73, 31.73, 29.24, 28.95, 27.62, 22.72, 14.18 ppm.

^1^H NMR (300 MHz, CDCl_3_) *δ* = 8.02 (d, *J* = 8.7 Hz, 1H), 7.89–7.40 (bs), 7.15 (d, *J* = 8.7 Hz, 2H), 6.91 (d, *J* = 8.7 Hz, 2H), 6.88–6.83 (m, 2H), 3.78 (s, 3H), 2.58–2.49 (m, 2H), 1.75–1.55 (m, 2H), 1.36–1.10 (m, 8H), 0.94–0.76 (m, 3H) ppm.

### 2.2. Mouse Study

#### 2.2.1. Preparation of Experimental Diets

The basic diet used for preparation of the experimental diets was C1000 (Altromin, Germany), a standard maintenance rodent diet free of phytoestrogens and with all polysaccharides derived from corn starch. Three experimental diets were prepared; a diet enriched with 2% cholesterol, a diet enriched with 2% cholesterol and 1000 mg/kg formononetin (3.7 mmol/kg), and a diet enriched with 2% cholesterol and 1300 mg/kg C7F (3.6 mmol/kg). For pelleting 2% gelatin, 0.5% magnesium-stearate and 5% talcum were added to the diets. For composition of the diet See Supplementary Table 1 in Supplementary material available online at http://dx.doi.org/10.1155/2013/926942.

#### 2.2.2. Animals and Study Design

All animal studies were performed in accordance with the Council of Europe Convention ETS 123, in which the principles are equivalent to the PHS Policy on Humane Care and Use of Laboratory Animals. The study was approved by the Danish Animal Experimentation Inspectorate (License no. 2007-561-1434). All mice were housed in type III makrolon cages (Tecniplast, Italy) with aspen bedding and environmental enrichment (Tapvei Oy, Finland). All mice were housed under environmentally controlled conditions with an alternating 12-hour light : dark cycle with access to food and water *ad libitum* except when food was withheld for the experimental protocols described in the following. Food and water were changed several times weekly. C57BL/6JBomTac mice, purchased from Taconic (Denmark), were selected for the study as it is one of the mostly used mouse strains within this field due to its susceptibility to diet-induced obesity, type 2 diabetes, and low-grade inflammation [21]. The mice were approximately four weeks old when recruited to the study as we wanted to study the effects in early life as obesity problems are often established when growing up. 

*Experiment  1*. 126 male mice were housed in groups of five mice. The mice were randomly divided into five groups and allowed one week of acclimatisation where they were fed the cholesterol-free C1000 diet. After this period, one group (*n* = 30) was euthanized and the remaining mice were fed the cholesterol diet for five weeks to induce hypercholesterolemia. After this period, another group (*n* = 23) was euthanized and the remaining mice were fed the experimental diets (cholesterol (*n* = 25), formononetin (*n* = 23), or C7F (*n* = 25)) for additionally three weeks before they were euthanized. The group size was calculated based upon serum cholesterol levels from a previous study on isoflavones in mice [22], in which a difference in relation to isoflavone feeding was approximately 5% with a standard deviation of approximately 6%. Thus a power calculation setting the power to 0.9 showed that a difference could be shown with 25 mice and *P* < 0.01. Body weight was measured weekly during the entire period. 

*Experiment  2.* 32 male mice were housed in groups of four mice. The mice were randomly divided into four groups (*n* = 8) and allowed one week of acclimatisation where they were fed the cholesterol-free C1000 diet. The group size was calculated on the basis of a previous study on gene expression in relation to daidzein feeding [9], in which the relevant gene expressions were at least 50% different between the groups, and no standard deviation was more than 25%. Thus a power calculation setting the power to 0.9 showed that a difference could be shown with 8 mice and *P* < 0.01. One group was fed the cholesterol-free C1000 diet (chow) for the whole experiment. The other three groups were fed the cholesterol-enriched diet for five weeks before initiation of experimental period with feeding of cholesterol, formononetin, or C7F for additionally five weeks. Body weight and food intake were measured weekly during the entire period. 

For termination of both studies, the mice were deprived of food for 12 hours. The mice were anesthetised with a mixture of Hypnorm (Vetapharma, UK) and Dormicum (Roche, Denmark) as previously described [23]. After loss of reflexes, blood was collected from the retroorbital sinus into heparinised tubes, and the mice were euthanized by cervical dislocation. In Experiment  1, the livers were removed, and a slice from each liver was transferred to RNA later and kept at −20°C. Caecum samples were collected aseptically and immediately frozen at −80°C until use. In Experiment  2, white adipose tissues (WAT) (inguinal WAT (iWAT), epididymal WAT (eWAT), intrascapular brown adipose tissue (iBAT), and liver were dissected, and half was freeze clamped and frozen at −80°C and the other half was fixed in 4% paraformaldehyde in phosphate buffer and later dehydrated and embedded in paraffin.

### 2.3. Oral Glucose Tolerance Test

An oral glucose tolerance test was performed in Experiment  1 prior to the initiation of the experimental diets and again at termination of the experimental period. The mice were fasted overnight, blood was collected from the tail vein as previously described [24] at time points −30, 0, 30, 60, 120, and 180 minutes, and glucose was monitored immediately on a FreeStyle Mini glucometer (Hermedico, Denmark) as previously described [25]. After the first two blood samples at *t* = 0, each mouse was dosed p.o. with 2 g/kg glucose (500 g/L Glucose SAD infusion solution, Veterinary Pharmacy, University of Copenhagen, Denmark).

### 2.4. DXA Scan

Prior to euthanasia in Experiment  1, Dual Energy X-ray Absorptiometry (DXA) scan (GE Lunar Prodigy, General Electric, WI, USA) was performed with the scanner running the small animal software from the same manufacturer. The anesthetised mice were placed at specific marks on a piece of carton to make sure that all animals were similarly arranged in the scanner. The carton alone was evaluated prior to the first scan with a satisfying result (no measureable values). Body weight, body fat percentage, fat mass, bone mineral content, and bone mineral density were determined.

### 2.5. Blood Sampling and Analysis of Plasma Lipids

Plasma was harvested after centrifugation at 3000 g at room temperature for 10 min and stored at −80°C until analysed. From Experiment  1, total plasma cholesterol, HDL-cholesterol, LDL-cholesterol, and triglycerides were measured enzymatically and photometrically on ABXPentra400 (Horiba Group, France) using 120 *μ*L of plasma. From Experiment  2, plasma levels of alanine transaminase (ALT) and aspartate transaminase (AST) were analysed using Biovision kits (AH Diagnostics, Denmark). Samples were run in duplicates for all analyses.

### 2.6. Gene Expression

*Samples from Experiment  1*. Total RNA was isolated from liver slices using the Nucleospin Kit (Macherey Nagel, Germany) according to manufacturer's protocol. The quality of the RNA was assessed (all 260/280 > 2 and all 260/230 > 1.7). The reverse transcription and PCR were set up using RT^2^ First-strand Kit and RT^2^ SYBR Green qPCR Master Mix from SABiosciences (Tebu-bio, Denmark). The reactions were set up according to the Kit manuals. The gene expression analysis was carried out using Mouse Drug Metabolism array plates from SA Biosciences. Three array plates were set up for each group of animals (seven animals per plate). For each array, 1.5 *μ*g total RNA was used, 0.21 *μ*g of total RNA from each animal. In the mouse drug metabolism array, gene expression of 84 genes involved mainly in phase I and phase II metabolism can be analysed (Supplementary Table 2). The array also includes negative and positive control and the following 5 housekeeping genes: *β*-glucuronidase, hypoxanthine guanine phosphoribosyl transferase 1, heat shock protein 90 *α*, glyceraldehyde-3-phosphate dehydrogenase, and *β*-actin in order to calculate expression changes. 

*Samples from Experiment  2*. Total RNA was purified from eWAT, iWAT, iBAT, and liver using Trizol (Invitrogen, Denmark) and RNA concentration was measured on a Nanodrop (Thermo Scientific, Denmark). cDNA was synthesised with RevertAid (Fermentas, Germany) according to manufacturer's instructions. Reactions were diluted with 120 *μ*L of water and frozen at −80°C until analysed on Roche LightCycler 480 (Roche). cDNA was analysed in duplicates in 20 *μ*L reactions containing SYBR Green Mastermix (Roche), 3 *μ*L of diluted cDNA, and 300 nM of each primer. Reaction mixtures were denaturated at 95°C for 2 min followed by 40 cycles of 95°C/15 s, 60°C/15 s, 72°C/20 s. Data was analysed using Roche Lightcycler software and the ΔΔCt method and normalised to 18S ribosomal RNA. Primers for RT-PCR were purchased from TAG Copenhagen (Denmark) (Supplementary Table 3). 

### 2.7. Gut Microbiota Composition Analysis

DNA was extracted from the ceacum samples from Experiment  1 using the QIAamp DNA Stool Mini Kit (Qiagen, Germany) according to the manufacturer's instructions and stored at −40°C until analysis during which the V3 region of the 16S rRNA gene was amplified by PCR using the following universal primer set PRBA338f and PRUN518r (5′-C GCC CGC CGC GCG CGG CGG GCG GGG CGG GGG CAC GGG GGG ACT CCT ACG GGA GGC AGC AG-3′ and 5′-ATT ACC GCG GCT GCT GG-3′) [26] (Eurofins MWG Operon, Germany). All reactions were carried out in a 50 *μ*L volume containing 1.25 U HotMaster Taq DNA Polymerase (5 Prime, Germany), 5 *μ*L 10× HotMaster Taq Buffer with 2.5 mM MgCl_2_ (5 Prime)_, _100 ng DNA, 10 pmol of each primer, 0.3 mM dNTP (Bioline, Germany), and 1 *μ*g BSA (Sigma-Aldrich). The PCR reaction was performed on a Robocycler Thermoblock (Stratagene, Denmark). Initial denaturation was done at 95°C for 5 minutes, and amplification was carried out using 30 cycles each including denaturation at 95°C for 30 seconds, annealing at 60°C for 30 seconds, and extension at 72°C for 40 seconds, followed by a final elongation step at 72°C for 10 minutes. A final product length of approximately 230 bp was checked by electrophoresis on a 2% agarose gel, stained with Ethidium Bromide (Bio-Rad, CA, USA). PCR amplicons were analysed by DGGE using the INGENYphorU-2 system according to the manufacturer's instructions (INGENY, The Netherlands). The acrylamide concentration in the gel was 9% and the linear denaturation gradient was 30% to 65% (100% denaturant corresponds to 7 M urea and 40% deionized formamide). Before loading, 35 *μ*L PCR product was mixed with 6 *μ*L 6× loading dye. In addition to the samples analysed, an in-house standard PCR product was loaded, allowing accurate alignment of lanes and bands within and between gels. Electrophoresis was performed in 0.5× TAE (1× TAE corresponds to 40 mM Tris-acetate, 1 mM EDTA, pH 8.0), at 60°C for 16 hours at 120 Volt. Staining was performed with a 1 : 10000 SYBR Gold staining solution (Invitrogen, OR, USA) in 1× TAE for 1 hour and photographed with UV transillumination (302 nm) using a Kodak EDAS 290 system (Eastman Kodak).

### 2.8. Histology

From Experiment  2, sections of paraffin-embedded adipose tissue and liver were cut into 3 *μ*m thick slices and stained with haematoxylin and eosin according to standard procedures. 

### 2.9. Triglyceride Measurements

From Experiment  2, total lipids were extracted from the liver using a modified version of the Bligh and Dyer protocol. In brief, 25 mg tissues was homogenised in potassium phosphate buffer, and lipids were extracted with chloroform:methanol (1 : 2). HCl was added, and the chloroform phase transferred to new tubes and evaporated under nitrogen. The extract was dissolved in LPL buffer (28.75 mM PIPES, 57.41 mM MgCl_2_·6H_2_O, 0.569 mg/mL BSA-FFA, 0.1% SDS) and analysed with a triglyceride kit (Zen-Bio, NC, USA). 

### 2.10. Statistics

Area under curve (AUC) was calculated from weighing and data from the oral glucose tolerance test. Three-dimensional principal component analysis (3D-PCA) based on DGGE data was carried out (Applied Maths). All quantitative data were tested for normality by Anderson-Darling test, compared in a general linear model with the settings group cage (group), and finally significant differences between groups were further evaluated comparing individual groups by an unpaired two-sample *t*-test. Software developed by SABiosciences (Tebu-bio) specifically for gene expression arrays was used to calculate the fold changes in gene expression for gene expression and *P* values in Experiment  1. In Experiment  1, all ANOVAs were performed by the software Minitab ver. 14 (Minitab, PA, USA). In Experiment  2, differences between the groups were analysed using the GLM procedure in SAS (SAS 9.3, SAS Institute). Data were considered statistically significant when *P* ≤ 0.05.

## 3. Results

### 3.1. Supplementation with C7F Decreased Body Weight Gain

**Figure 1:**
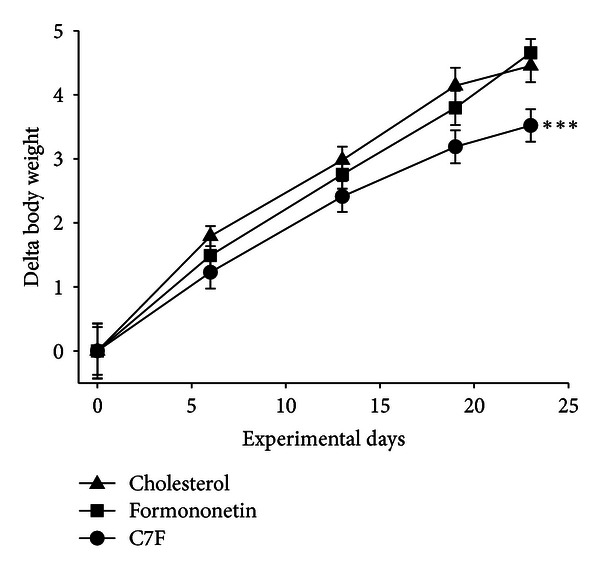
Body weight of cholesterol fed C57BL/6 mice (*n* = 23–25) supplemented with either formononetin or 2-heptyl-formonetin (C7F) for three weeks (Experiment  1). Graphs show mean ± SEM. *P ≤ 0.05.

 At the end of Experiment  1, mice fed cholesterol plus C7F weighed significantly less than the mice fed only cholesterol (*P* < 0.05). There were no weight differences between the mice fed only cholesterol and those fed cholesterol plus formononetin (Figure 1). The fat percentage was decreased in all three experimental groups after cholesterol feeding and after the experimental period compared to the initial acclimatisation period. However, there were no differences among the three experimental groups (Table 1). Bone mineral concentration and bone mineral density increased significantly from the initial acclimatisation period over the cholesterol induction period till the experimental period, but there were no differences among the experimental groups (Table 1).

Difference in weight development can be due to differences in gut microbiota [27]. However, even though there was a significant clustering in gut microbiota composition in relation to feeding on the *y*- and *z*-axis of the PCA-plot this was mainly due to caging (Supplementary Figure 1). 

### 3.2. The Cholesterol-Enriched Diet Increased Plasma Levels of Total Cholesterol and HDL-Cholesterol and Decreased Plasma Levels of Triglycerides

The cholesterol-enriched diet was expected to elevate the plasma level of cholesterol. However, although total plasma cholesterol increased after the first five weeks of cholesterol feeding, the difference was not significant. Yet, after the experimental period, the plasma level of total cholesterol was significantly higher for mice fed cholesterol compared to the mice euthanized before initiation of cholesterol feeding. Surprisingly, the plasma level of HDL-cholesterol was significantly increased, and the plasma level of triglycerides was decreased in mice fed cholesterol compared to mice euthanized before initiation of cholesterol feeding. Cholesterol feeding did not affect LDL-cholesterol (Table 2).

### 3.3. Supplementation with C7F Increased Plasma Cholesterol

Mice fed C7F had significantly higher plasma levels of total cholesterol and HDL-cholesterol than mice fed cholesterol or formononetin. Furthermore, mice fed formononetin had increased plasma level of triglycerides compared to mice fed cholesterol. There were no differences between the experimental groups with respect to LDL-cholesterol (Table 2).

### 3.4. Formononetin and C7F Did Not Affect Glucose Tolerance

**Figure 2:**
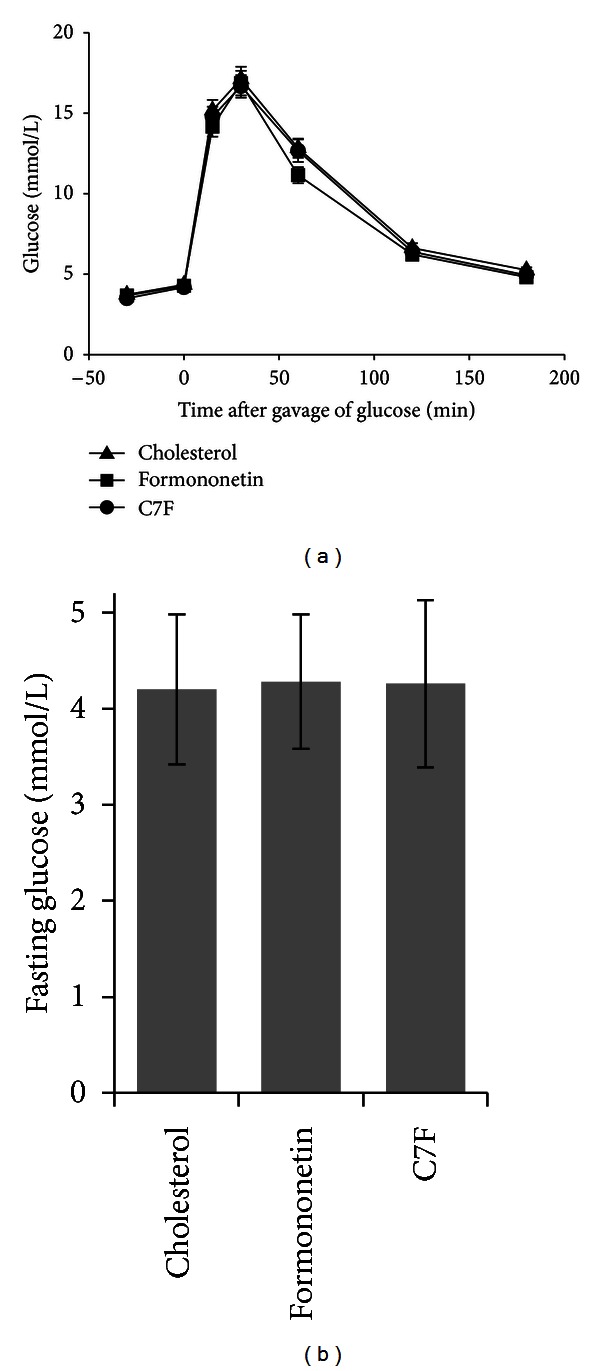
Glucose homeostasis of cholesterol fed C57BL/6 mice (*n* = 23–25) supplemented with either formononetin or 2-heptyl-formononetin (C7F) for three weeks (Experiment  1). (a) Glucose clearance assessed by oral glucose tolerance test (2 g/kg glucose). (b) Fasting plasma glucose concentration. Graphs show mean ± SEM.

Isoflavones have been reported to improve glucose uptake *in vitro* and glucose tolerance *in vivo* [28, 29]. However, mice fed cholesterol diet supplemented with either formononetin or C7F did not differ in glucose tolerance as monitored by an oral glucose tolerance test (Figure 2(a)) or in fasting glucose levels (Figure 2(b)) (Experiment  1) compared to control mice fed only the cholesterol diet.

### 3.5. C7F Upregulated the Expression of *Gstm1*

Isoflavones have been shown to affect phase I and II metabolism of drugs in the liver [30]. Scatter plots of the liver gene expression in Experiment  1 showed that *Gstm1 *(glutathione S-transferase Mu 1) was significantly upregulated 2.4 times in mice fed cholesterol plus C7F compared to those fed only cholesterol. Furthermore, the expression of *Cyp11b2* (aldosterone synthase) was 12.9 times upregulated in mice fed C7F compared to formononetin (Supplementary Figure 2). 

### 3.6. Formononetin and C7F Induced Hepatic Steatosis

To further asses the effects of formononetin and C7F on lipid metabolism in liver and adipose tissues, a second experiment was carried out. There were no significant differences in weight development in this study perhaps because of the lower number of mice in each group (Supplementary Figure 3). Feed intake was measured weekly but showed no differences between the groups (Supplementary Figure 4). 

**Figure 3:**
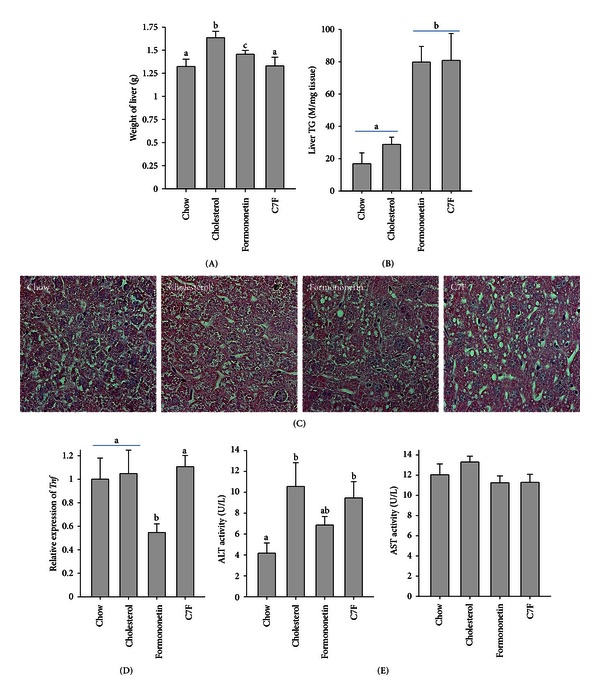
Development of hepatic steatosis in C57BL/6 mice fed chow, cholesterol, or cholesterol supplemented with formononetin or 2-heptyl-formonetin (C7F) for five weeks (Experiment  2). (A) Weight of liver (*n* = 8). (B) Triglyceride content in liver (*n* = 6). Total lipids were extracted from liver using a modified version of the Bligh and Dyer protocol, and the content of triglyceride were analysed with a commercial kit. (C) Liver sections stained with hematoxylin and eosin. (D) Hepatic gene expression of *Tnf* (tumour necrosis factor *α*) measured by RT-PCR. Data is normalised to 18S ribosomal RNA and presented relatively to the expression in chow (*n* = 6). (E) Plasma level of aspartate aminotransferase (AST) and alanine aminotransferase (ALT) (*n* = 6). Graphs show mean ± SEM. Different letters (a, b) denote significant difference (*P* ≤ 0.05) between the groups.

It is well-documented that genistein and daidzein protect against the development of hepatic steatosis in rodents fed high-fat diets [9, 10, 14, 15]. At termination of Experiment  2 weight of the liver was significantly increased in the mice fed cholesterol compared to the other groups. The liver weight was similar for chow and C7F fed mice but increased for mice fed formononetin (Figure 3(A)). Quantification of triglycerides in the liver showed no difference between mice fed chow and cholesterol. In contrast, there was a large increase in hepatic accumulation of triglycerides in the mice fed formononetin and C7F (Figure 3(B)). The development of hepatic steatosis was confirmed by visual examination of H&E stained sections of the livers (Experiment  2) revealing clear microvesicular structures presumably from fat vacuoles in mice fed C7F and formononetin (Figure 3(C)).

### 3.7. Formononetin Protected against Hepatic Inflammation and Dysfunction

The development of hepatic steatosis is often associated with hepatic inflammation and/or liver injury. Surprisingly, the expression of *Tnf* (tumour necrosis factors *α*) was similar in mice fed chow, cholesterol, and C7F but decreased in mice fed formononetin (Figure 3(D)), suggesting less hepatic inflammation in formononetin fed mice. Similarly, the plasma level of ALT was increased in mice fed cholesterol and C7F compared to mice fed chow, whereas there was no increase for mice fed formononetin (Figure 3(E)). This indicates increased damage to the hepatocytes in mice fed cholesterol and C7F but not in mice fed formononetin. There was no difference in the plasma level of AST (Figure 3(E)).

### 3.8. Formononetin and C7F Decreased Lipogenesis, *β*-Oxidation, and Lipoprotein Metabolism

To investigate possible routes by which formononetin and C7F might induce hepatic steatosis, we measured hepatic expression of genes involved in lipogenesis, *β*-oxidation, and lipoprotein metabolism (Experiment  2).

**Figure 4:**
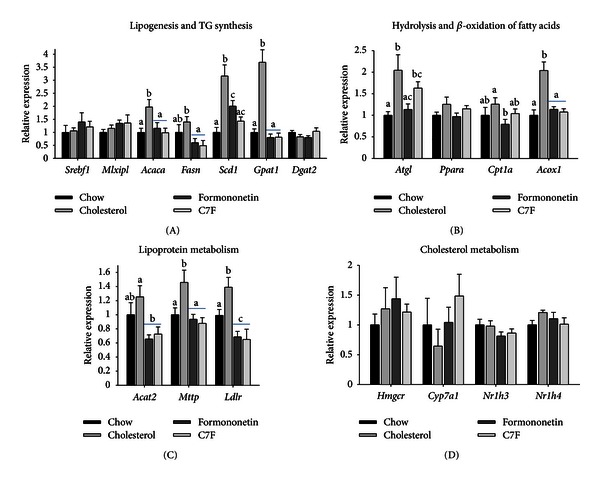
Hepatic gene expression measured by RT-PCR in C57BL/6 mice fed chow, cholesterol, or cholesterol supplemented formononetin or 2-heptyl-formonetin (C7F) for five weeks (Experiment  2). (A) Genes involved in lipogenesis (*Srebf1 *(sterol regulatory element-binding protein-1c), *Mlxipl* (carbohydrate response element binding protein), *Acaca *(acyl-CoA carboxylase 1), *Fasn* (fatty acid synthase), and *Scd1* (stearoyl-CoA desaturase 1)) and synthesis of triglycerides (*Gpam* (glycerol phosphate acyltransferase) and *Dgat2* (diglyceride acyltransferase 2)). (B) Genes involved in hydrolysis and beta-oxidation of fatty acids, *Atgl* (adipose triglyceride lipase), Ppara (peroxisome proliferator-activated receptor *α*), *Cpt1a* (carnitine palmitoyltransferase 1a), and *Acox1* (acyl CoA oxidase). (C) Genes involved in lipoprotein metabolism, *Acat2* (acetyl-CoA acetyltransferase), *Mttp* (microsomal triglyceride transfer protein), and *Ldlr* (low-density lipoprotein receptor). (D) Genes involved in cholesterol metabolism, *Hmgcr* (3-hydroxy-3-methyl-glutaryl-CoA reductase), *Cyp7a1* (cholesterol 7 alpha-hydroxylase), *Nr1 h3* (liver X receptor), and *Nr1 h3* (farnesoid X receptor). Data is normalised to 18S ribosomal RNA and presented relative to the expression in chow (*n* = 6). Graphs show mean ± SEM. Different letters (a, b, c) denote significant difference (*P* ≤ 0.5) between the groups.

Surprisingly the expressions of *Acaca* (acyl-CoA carboxylase-1) and *Fasn* (fatty acid synthase), the rate-limiting genes in lipogenesis, were significantly upregulated in cholesterol fed mice compared to chow but similar to chow for mice fed formononetin and C7F. The pattern was the same for *Scd1* (stearoyl-CoA desaturase), the rate-limiting gene in the synthesis of monounsaturated fatty acids, although the expression was increased in formononetin compared to chow but not as much as in cholesterol-fed mice. There were no differences in the expressions of the lipogenic transcription factors *Srebf1* (sterol regulatory element-binding protein-1c) and *Mlxipl* (MLX interacting protein-like or carbohydrate response element binding protein). The genes *Gpam* (glycerol phosphate acyltransferase) and *Dgat2* (diglyceride acyltransferase 2) are both central to the synthesis of triglycerides. Compared to chow-fed mice, the expression of *Gpam* was upregulated in mice fed cholesterol but similar in mice fed formononetin and C7F. There was no difference between the groups for the expression of *Dpat2* (Figure 4(A)).

Compared to mice fed chow, the expression of the lipolytic gene *Atgl* (adipose triglyceride lipase) was upregulated in mice fed cholesterol and C7F but not affected in mice fed formononetin. There was no difference in the expression of *Ppara* (peroxisome proliferator-activated receptor *α*), a transcription factor involved in catabolism of fatty acids. The expression of *Acox1* (acyl-CoA oxidase), involved in peroxisomal *β*-oxidation, was upregulated in cholesterol fed mice compared to the three other groups whereas the expression of *Cpt1a* (carnitine palmitoyl-CoA transferase-1a), involved in mitochondrial *β*-oxidation, was the same in all three groups compared to chow, although the expression was decreased in mice fed formononetin compared to cholesterol (Figure 4(B)). 

The expression of *Acat2* (acetyl-CoA acetyltransferase 2), responsible for synthesis of cholesteryl esters, was similar for mice fed cholesterol and chow but downregulated in mice fed formononetin and C7F. The expressions of *Mttp *(microsomal triglyceride transfer protein), which controls the assembly of lipoproteins, and *Ldlr* (low-density lipoprotein receptor), which mediates endocytosis of ApoB-containing lipoproteins, were both increased in mice fed cholesterol compared to chow but similar chow-fed mice and mice fed formononetin and C7F (Figure 4(C)).

As the mice were fed a cholesterol-enriched diet, it seemed likely that the metabolism of cholesterol could be affected. However, there was no difference in the level of genes central in cholesterol metabolism (*Hmgcr* (3-hydroxy-3-methylglutaryl-Coenzyme A reductase), *Cyp7a1* (cholesterol 7 alpha-hydroxylase), *Nr1h3* (liver X receptor *α*), and *Nr1h4* (farnesoid X receptor)) (Figure 4(D)).

### 3.9. C7F Increased Lipogenic and Lipolytic Gene Expression in iWAT

**Figure 5:**
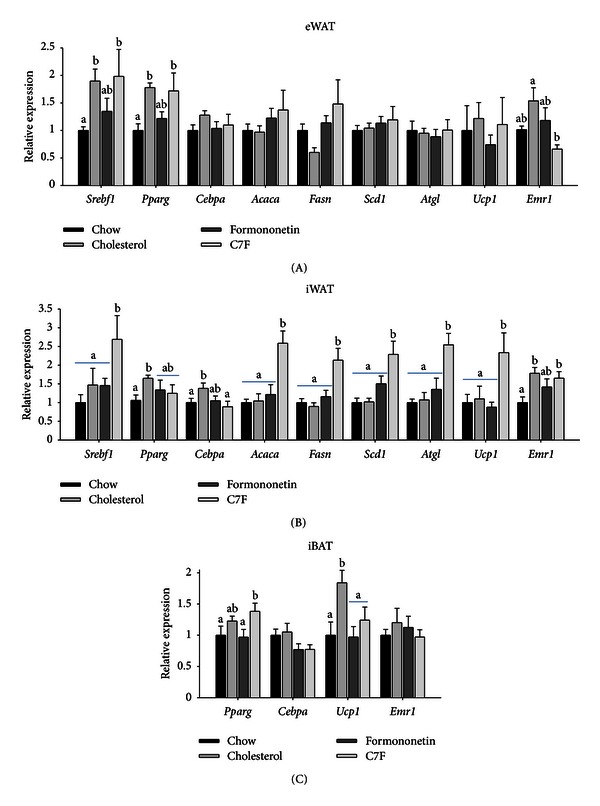
Adipocyte gene expression in C57BL/6 mice fed chow, cholesterol, or cholesterol supplemented formononetin or 2-heptyl-formonetin (C7F) for five weeks (Experiment  2). Gene expression measured by RT-PCR of *Srebf1 *(sterol regulatory element-binding protein-1c), *Pparg* (peroxisome proliferator-activated receptor *γ*), *Cebpa* (CCAAT/enhancer-binding protein *α*), *Acaca *(acyl-CoA carboxylase 1), *Fasn* (fatty acid synthase), *Scd1* (stearoyl-CoA desaturase 1), *Atgl* (adipose triglyceride lipase), *Ucp1* (uncoupling protein 1), and *Emr1* (F4/80) in (A) eWAT, (B) iWAT, and (C) iBAT measured by RT-PCR. Data is normalised to 18S ribosomal RNA and presented relative to the expression in chow (*n* = 6). Graphs show mean ± SEM. Different letters (a, b) denote significant difference (*P* ≤ 0.5) between the groups.

We also examined gene expression in the adipose tissues (Experiment  2). Of interest, the expressions of *Srebf1* and *Pparg* (PPAR *γ*), master regulators of lipogenesis, were upregulated in eWAT in mice fed cholesterol and C7F compared to mice on chow (Figure 5(A)). Furthermore, the expressions of *Srebf1*, *Acaca*, *Fasn*, and *Scd1* as well as *Atgl* were upregulated in iWAT from mice fed C7F compared to the three other groups (Figure 5(B)). The expression of *Ucp1* (uncoupling protein-1), essential for nonshivering thermogenesis, was upregulated in iBAT from cholesterol-fed mice compared to the three other groups and in iWAT from mice fed C7F compared to the three other groups (Figures 5(B), and 5(C)). Also of interest, the expression of *Emr1* (EGF-like module containing, mucin-like, hormone receptor-like sequence 1 or F4/80), a macrophage marker, was increased in eWAT from mice fed cholesterol and C7F, but compared to cholesterol-fed mice the expression was down-regulated in both eWAT and iWAT in mice fed formononetin (Figures 5(A) and 5(B)).

Visual examination of H&E stained sections of eWAT, iWAT, and iBAT showed no differences in size of the adipocytes between the groups (data not shown) (Experiment  2).

## 4. Discussion

Supplementation with formononetin or C7F to C57BL/6J mice fed a cholesterol-enriched diet had limited effects on body weight, body composition, and glucose tolerance. However, C7F increased the serum level of total cholesterol and HDL-cholesterol. More importantly, formononetin and C7F induced hepatic steatosis by affecting adipocyte and hepatic gene expression, although hepatic gene expression of *Tnf* was decreased by formononetin.

Studies with genistein and daidzein using doses comparable to this study show a substantial decrease in body weight and fat mass [9–11] and improved glucose tolerance [28]. However, genistein and daidzein have been supplemented to mice fed high-fat diets and thus getting considerably obese which could explain contradictory results in the present study.

Surprisingly, formononetin and C7F induced hepatic steatosis. Increased lipogenesis and/or decreased *β*-oxidation promote the development of hepatic steatosis [31]. Hepatic gene expression suggested decreased peroxisomal *β*-oxidation but also decreased lipogenesis and decreased triglyceride assembly in mice fed formononetin and C7F compared to cholesterol suggesting overall decreased hepatic lipid metabolism. In mice fed formononetin, the expression of *Acox1* was slightly decreased, correlating with decreased expression of *Atgl*. Mice with liver-specific deletion of *Atgl* have severe hepatic steatosis but normal plasma levels of glucose, triglycerides, and cholesterol [32]. Thus, deceased lipolysis and *β*-oxidation could partly explain the development of hepatic steatosis, especially for mice fed formononetin, although decreased lipogenic gene expression could counteract this effect. In agreement with our results, genistein and daidzein decrease lipogenic gene expression [14, 33], whereas the expression of genes involved in *β*-oxidation has been decreased in some studies [34, 35] but not affected in others [14, 15]. This suggests that other factors are involved in the increase in hepatic steatosis in this study.

The development of hepatic steatosis can also be caused by decreased export of fatty acids from the liver due to deregulated lipoprotein metabolism. Microsomal triglyceride transfer protein (MTTP) deficient mice have reduced plasma triglycerides levels but develop hepatic steatosis without insulin resistance and inflammation [36]. Similarly, low-density lipoprotein receptor (LDLR) deficient mice also develop hepatic steatosis [37]. Thus, although we did not observe a decrease in plasma triglycerides, decreased expression of *Mttp* and *Ldlr* could be a major cause of the development of hepatic steatosis in mice fed formononetin and C7F. The effects on lipoprotein metabolism by formononetin and C7F in this study are to a large extent supported by a study in HepG2 cells by Borradaile et al. [38]. In their study genistein and daidzein decreased apolipoprotein B secretion through decreased MTTP activity and mRNA expression and decreased acetyl-Coenzyme A acetyltransferase activity. However, they report increased expression of *Ldlr*. Interestingly, in this study, genistein also increased triglyceride mass in the cells. 

Other isoflavones have been shown to decrease lipogenic gene expression in adipocytes both *in vitro* [39–41] and *in vivo* [10, 14]. In 3T3-L1, preadipocytes lower concentrations of C7F increase lipid accumulation, whereas high concentrations decrease lipid accumulation (manuscript in preparation). This response is similar to genistein both *in vitro* (unpublished results) and *in vivo* [11]. Based on this study, it is not possible to conclude why C7F, in contrast to other isoflavones, increased lipogenic gene expression *in vivo*. Both genistein and daidzein have been shown to induce lipolysis [40–42]. Increased expression of *Atgl* by C7F suggests increased lipolysis in iWAT which could explain why there is no increase in fat mass despite increased lipogenic gene expression. Moreover, this could imply a flux of fatty acids from iWAT to the liver. 

Despite increased accumulation of triglycerides in the liver, formononetin decreased level of plasma ALT and hepatic expression of *Tnf* indicating diminished liver damage and lower hepatic inflammation. Furthermore, the decreased expression of *Emr1* in iWAT and eWAT suggests lower infiltration of macrophages in mice fed formononetin. Isoflavones are known to be anti-inflammatory compounds, and other studies also report decreased plasma levels of AST, ALT, and tumour necrosis factor *α* [6, 13, 43] and decreased adipocyte and hepatic expression of *Tnf* [10, 43]. Accumulation of lipids in hepatocytes impairs the oxidative capacity of the mitochondria thereby increasing the generation of reactive oxygen species. Reactive oxygen species trigger lipid peroxidation, release of inflammatory cytokines, and cell death and thereby induce hepatic inflammation and fibrosis [44]. Some of the effects of isoflavones have been attributed to the antioxidative capacity. Yet formononetin has a lower antioxidative capacity than genistein and daidzein [45]. This could partly explain why formononetin and C7F did not prevent hepatic steatosis. Still, the lower levels of plasma ALT and hepatic expression of *Tnf* in formononetin fed mice compared to C7F fed mice could be due to a higher antioxidative capacity of formononetin than C7F.

In contrast to our results, a range of studies show that plasma total cholesterol, LDL-cholesterol, and triglycerides are decreased by genistein [10, 12, 13], daidzein [9], and formononetin [46]. However, LDL is a difficult parameter in mice, as the levels are normally very low and the variation still substantial [9]. Conversely, the effects on HDL-cholesterol vary; some studies show upregulation [10, 13, 28], one study shows downregulation [12], and two studies show no effect [14, 46]. Still, based on the development of hepatic steatosis and dysregulated lipid and lipoprotein metabolism, it seems plausible that plasma lipid composition was dysregulated in mice fed formononetin and C7F. The increased plasma level of total cholesterol in mice fed C7F seemed to be caused by a rise in HDL-cholesterol. When LDL circulates in the blood, it can slowly build up in the inner walls of the arteries forming plaques leading to atherosclerosis. In contrast, HDL tends to carry cholesterol away from the arteries and back to the liver. Thus, the increase in HDL-cholesterol could protect against cardiovascular diseases. However, in contrast to humans HDL is the essential cholesterol fraction of mice, whereas the level of LDL-cholesterol is minimal [47]. Therefore, it can be difficult to affect the level of LDL-cholesterol in mice and to extrapolate data on lipid profiles from mice to humans. 

Our study suggests that even though bioactive compounds have very similar structures, the biological actions can be very different. It is a possibility that the different actions of formononetin and C7F reported in this study are specifically due to the use of a cholesterol-enriched diet instead of chow and high-fat diets used in other studies. It would therefore be interesting to assess the metabolic effects of genistein and daidzein using other diets like a cholesterol-enriched diet to see if this affects the health benefits associated with these compounds. 

## 5. Conclusions

In conclusion, we showed that supplementation with formononetin and C7F to C57BL/6J mice fed a cholesterol-enriched diet induced hepatic steatosis affecting adipocyte and hepatic gene expression. Of note, in spite of the hepatosteatotic phenotype, formononetin, but not C7F, decreased markers of inflammation and liver injury.

## Supplementary Material

Supplementary Table 1: Composition of the diets.
Supplementary Table 2: A list of functional gene groupings included in the gene expression array analysis of phase I and II enzymes on C57BL/6 mice fed cholesterol supplemented formononetin or 2-heptyl formononetin (CF7) for three weeks (experiment 1).
Supplementary Table 3: Primer sequences.
Supplementary Figure 1: Clustering of gut microbiota from C57BL/6 mice fed formononetin or 2-heptyl formononetin (C7F) (experiment 1) (n=23-25). Three-dimensional principal component analysis (3D-PCA) based on denaturation gradient gel electrophoresis data. Black circle: cholesterol, red square: formononetin, green square: C7F. The clustering was significant on the Y-axis and the Z-axis. On the Y-axis C7F fed mice clustered significantly differently from mice fed cholesterol and the formononetin. Also the formononetin fed mice clustered significantly differently from mice fed cholesterol. On the Z-axis the C7F fed mice clustered borderline differently from mice fed cholesterol.
Supplementary Figure 2: Gene expression of phase I and II metabolites (experiment 1). Scatter plot of liver gene expression (n=23-25). Gene expression in A mice fed chow compared to formononetin, B chow compared to C7F and C formononetin compared to C7F.
Supplementary Figure 3: Body weight of C57BL/6 mice fed chow, cholesterol or cholesterol supplemented formononetin or 2-heptyl-formonetin (C7F) for five weeks (experiment 2) (n=8). Graph shows mean ± SEM.
Supplementary Figure 4: Total feed intake in C57BL/6 mice fed chow, cholesterol or cholesterol supplemented formononetin or 2-heptyl-formonetin (C7F) for five weeks (experiment 2) (n=8).
Graphs shows mean ± SEM.

## Figures and Tables

**Table 1:** Body composition as shown by DXA scans of C57BL/6 mice before cholesterol feeding, after five weeks of initial cholesterol feeding and after three additional weeks where the mice were fed the cholesterol-enriched diet supplemented with either formononetin or 2-heptyl-formonetin (C7F) (Experiment  1). Data show mean ± SEM. Different letters (a, b, c) denote significant difference between the groups.

	Fat percentage	Body weight (g)	Fat mass (g)	Bone mineral concentration (g)	Bone mineral density (mg/cm^3^)
Before test period					

After acclimatisation (*n* = 30)	19.3 ± 7.8^a^	16.7 ± 2.0^a^	3.2 ± 1.8	0.3 ± 0.1^ab^	640 ± 9^a^
After initial cholesterol feeding (*n* = 23)	13.7 ± 6.8^b^	24.3 ± 1.3^b^	3.2 ± 1.7	0.3 ± 0.1^a^	802 ± 10^b^

After test period					

Cholesterol (*n* = 25)	14.8 ± 8.2^b^	28.0 ± 2.1^c^	4.2 ± 2.6	0.4 ± 0.07^b^	864 ± 6^c^
Cholesterol + formononetin (*n* = 23)	13.1 ± 9.6^b^	27.8 ± 2.3^abc^	3.6 ± 2.8	0.4 ± 0.11^ab^	839 ± 9^bc^
Cholesterol + C7F (*n* = 24)	14.5 ± 5.5^b^	26.1 ± 2.2^d^	3.2 ± 1.7	0.4 ± 0.08^ab^	825 ± 10^bc^

**Table 2:** Plasma lipid profiles of C57BL/6 mice before cholesterol feeding, after five weeks of initial cholesterol feeding and after three additional weeks where the mice were fed the cholesterol-enriched diet supplemented with either formononetin or 2-heptyl-formonetin (C7F) (Experiment  1). Data show mean ± SEM. Different letters (a, b, c, d) denote significant difference (*P* ≤ 0.05) between the groups.

	Total cholesterol (mmol/L)	HDL (mmol/L)	LDL (mmol/L)	Triglycerides (mmol/L)
Before test period				

After acclimatisation (*n* = 30)	3.12 ± 0.20^a^	1.39 ± 0.10^a^	0.32 ± 0.05	1.95 ± 0.55^a^
After initial cholesterol feeding (*n* = 23)	3.22 ± 0.26^ab^	1.40 ± 0.11^ab^	0.35 ± 0.07	1.13 ± 0.30^ab^

After test period				

Cholesterol (*n* = 25)	3.32 ± 0.25^bc^	1.46 ± 0.15^b^	0.29 ± 0.06	0.95 ± 0.27^c^
Cholesterol + formononetin (*n* = 23)	3.43 ± 0.27^c^	1.44 ± 0.16^ab^	0.34 ± 0.14	1.16 ± 0.29^b^
Cholesterol + C7F (*n* = 25)	3.83 ± 0.54^d^	1.78 ± 0.18^c^	0.28 ± 0.10	1.03 ± 0.39^bc^
