# Supplementary material for: 2-Heptyl-Formononetin Increases Cholesterol and Induces Hepatic Steatosis in Mice
Source: Biomed Res Int. 2013 Apr 29;2013:926942. doi: 10.1155/2013/926942 (PMC3657407; doi:10.1155/2013/926942)

Supplementary data

Supplementary Table 1: Composition of the diets.

	**C1000**	**Cholesterol**	**Cholesterol + Formononetin**	**Cholesterol + C7F**
**Energy, kcal/kg**	3518	3444	3444	3444
**Crude protein, g/kg**	176	176	176	176
**Crude fat, g/kg**	50.8	66.6	66.6	66.6
**Crude fibre, g/kg**	40.5	31.0	31.0	31.0
**Disaccharides, g/kg**	111	111	111	111
**Polysaccharides, g/kg**	472	454	454	454
**Cholesterol, g/kg**	0	20	20	20
**Formononetin, mg/kg**	0	0	1000	0
**C7F, mg/kg**	0	0	0	1300

Supplemental Table 2: A list of functional gene groupings included in the gene expression array analysis of phase I and II enzymes on C57BL/6 mice fed cholesterol supplemented formononetin or 2-heptyl formononetin (CF7) for three weeks (experiment 1).

Drug Transporters:

Metallothioneins: Mt2, Mt3.

P-Glycoprotein Family: Abcb1a, Abcb1b, Abcb4, Abcc1, Gpi1.

Phase I Metabolising Enzymes:

P450 Family: Cyp11b2, Cyp17a1, Cyp19a1, Cyp1a1, Cyp1a2, Cyp27b1, Cyp2b10, Cyp2C29, Cyp2e1, Cyp4b1.

Phase II Metabolising Enzymes:

Carboxylesterases: Ces1, Ces2.

Decarboxylases: Gad1, Gad2.

Dehydrogenases: Adh1, Adh4, Adh5, Alad, Aldh1a1, Hsd17b1, Hsd17b2, Hsd17b3.

Glutathione Peroxidases: Gpx1, Gpx2, Gpx3, Gpx5, Gsta1, Gsta3, Gsta4, Gstm1, Gstm2, Gstm3, Gstm4, Gstm5, Gstp1, Gstt1, Gstz1, Lpo, Mpo.

Hydrolases: Ephx1, Faah, Fbp1.

Kinases: Hk2, Pklr, Pkm2.

Lipoxygenases: Alox12, Alox15, Alox5, Apoe.

Oxidoreductases: Blvra, Blvrb, Cyb5r3 (Dia1), Gpx1, Gpx2, Gsr, Mthfr, Nos3, Nqo1, Srd5a1, Srd5a2.

Paraoxonases: Pon1, Pon2, Pon3.

Sulfotransferases: Chst1, Gsta3, Gstm2, Gstm3, Gstm5, Gstp1, Gstt1, Mgst1, Mgst2, Mgst3.

Transferases: Nat1, Nat2, Comt, Ggt1.

Other Genes Related to Drug Metabolism: Abp1, Ahr, Arnt, Asna1, Gckr, Marcks, Smarcal1, Snn.

Supplemental Table 3: Primer sequences.

**Gene name**	**Trivial name**	**5' prime**	**3' prime**
Rn18S	Ribosomal 18S	GTA ACC CGT TGA ACC CCA TT	CCA TCC AAT CGG TAG TAG CG
Acat2	Acetyl-coA acetyltransferase 2	GGT GGA ACT ATG TGG CCA AGA	CCA GGA TGA AGC AGG CAT AGA
Acaca	Acetyl-coA carboxylase alpha	TGC TGC CCC ATC CCC GGG	TCG AAC TCT CAC TGA CAG G
Acox1	Acyl-Coenzyme A oxidase 1	GAT CAC GGG CAC TTA TGC G	CGA AGA TGA GTT CCA TGA CCC
Atgl	Adipose triglyceride lipase	CAT CTC CCT GAC TCG TGT TTC	CAA GTT GTC TGA AAT GCC GC
Cebpa	CCAAT/enhancer binding protein alpha	CAA GAA CAG CAA CGA GTA CCG	GTC ACT GGT CAA CTC CAG CAC
Cpt1a	Carnitine palmitoyltransferase 1a	TAC TGL TGT ATC GTC GCA CG	GAC GAA TAG GTT TGA GTT CCT CAC
Cpt1b	Carnitine palmitoyltransferase 1b	GAT CTG GGC TAT CTG TGT CCG T	ACG TTT GGA AGC TGT AGA GCA TG
Cyp7a1	Cholesterol 7 alpha-hydroxylase	AGC AAC TAA ACA ACC TGC CAG TAC TA	GTC CGG ATA TTC AAG GAT GCA
Dgat2	Diglyceride acyltransferase 2	GGG TCC AGA AGA AGT TCC AGA AG	CCC AGG TGT CAG AGG AGA AGA G
Emr1	EGF-like module containing, mucin-like, hormone receptor-like sequence 1	CTT TGG CTA TGG GCT TCC AGT	GCA AGG AGG ACA GAG TTT ATC TGT
Fasn	Fatty acids synthase	ATT GGT GGT GTG GAC ATG	CCCAGC CTT CCA TCT CCT G
Gpat1	Glycerol phosphate acyltransferase 1	GCT ATC ATG TCC ACC CAC ATT G	ACT TCC TCC TTC ATC ACA AAG AAG TC
Hmgcr	3-hydroxy-3-methylglutaryl-Coenzyme A Reductase	CCC TGA GTT TAG CCT TCC TTT TG	GCT TTC TTT GAG GTC ACG ACG G
Ldlr	Low density lipoprotein receptor	GCA TCA GCT TGG ACA AGG TGT	GGG AAC AGC CAC CAT TGT TG
Mlxipl	MLX interacting protein-like	CTT CTT CCG TTG CAC ATA CTG	GTT GCT ATG CCG GGA CAA GA
Mttp	Microsomal triglyceride transfer protein	CAA GCT CAC GTA CTC CAC TGA AG	TCA TCA TCA CCA TCA GGA TTC CT
Nr1h3	Nuclear receptor subfamily 1, group H, member 3	CCG ACA GAG CTT CGT CC	CCC ACA GAC ACT GCA CAG
Nr1h4	Nuclear receptor subfamily 1, group H, member 4	CCA ACC TGG GTT TCT ACC C	CAC ACA GCT CAT CCC CTT T
Ppara	Peroxisome proliferator-activated receptor alpha	AGA GAG GAC AGA TGG GGC TC	CGT TTG TGG CTG GTC AAG TT
Pparg2	Peroxisome proliferator-activated receptor gamma 2	ACAGCAAATCTCTGT TTTATGC	TGCTGGAGAAATCAAC TGTGG
Scd1	Stearoyl-CoA desaturase-1	ACA CCT GCC TCT TCG GGA TT	TGA TGG CCA GAG CGC TG
Srebf1	Sterol regulatory element-binding protein-1c	GGA GCC ATG GAT TGC ACA TT	GCT TCC AGA GAG GAG GCC AG
Tnf	Tumour necrosis factor	CCC TCA CAC TCA GAT CAT CTT CT	GCT ACG ACG TGG GCT ACA G
Ucp1	Uncoupling protein 1	AGCCGGCTTAATGACT GGAG	TCTGTAGGCTGCCCAA TGAAC

Supplementary figure legends and figures

Supplementary Figure 1: Clustering of gut microbiota from C57BL/6 mice fed formononetin or 2-heptyl formononetin (C7F) (experiment 1) (n=23-25). Three-dimensional principal component analysis (3D-PCA) based on denaturation gradient gel electrophoresis data. Black circle: cholesterol, red square: formononetin, green square: C7F. The clustering was significant on the Y-axis and the Z-axis. On the Y-axis C7F fed mice clustered significantly differently from mice fed cholesterol and the formononetin. Also the formononetin fed mice clustered significantly differently from mice fed cholesterol. On the Z-axis the C7F fed mice clustered borderline differently from mice fed cholesterol.

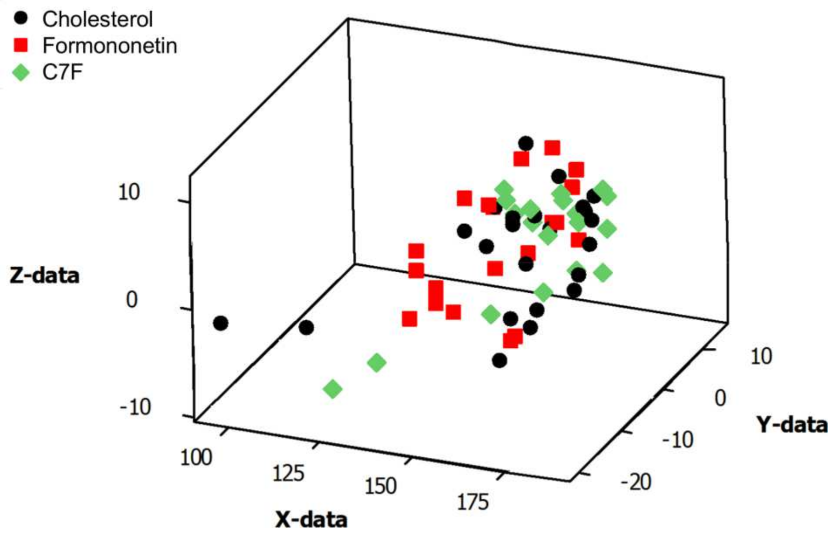

Supplementary Figure 2: Gene expression of phase I and II metabolites (experiment 1). Scatter plot of liver gene expression (n=23-25). Gene expression in **A** mice fed chow compared to formononetin, **B** chow compared to C7F and **C** formononetin compared to C7F.

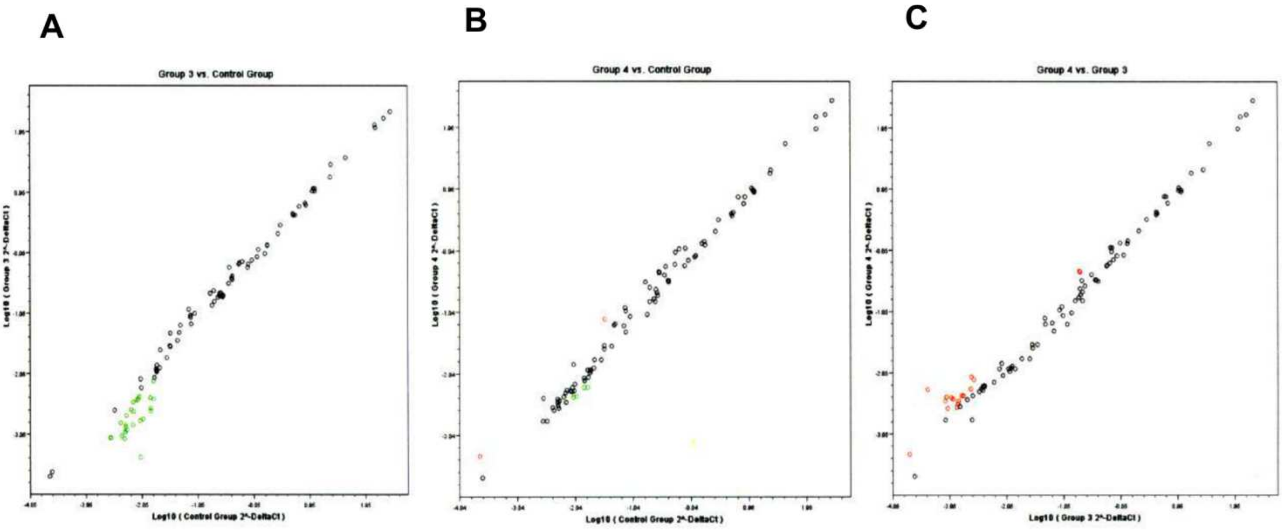

Supplementary Figure 3: Body weight of C57BL/6 mice fed chow, cholesterol or cholesterol supplemented formononetin or 2-heptyl-formonetin (C7F) for five weeks (experiment 2) (n=8). Graphs shows mean ± SEM.

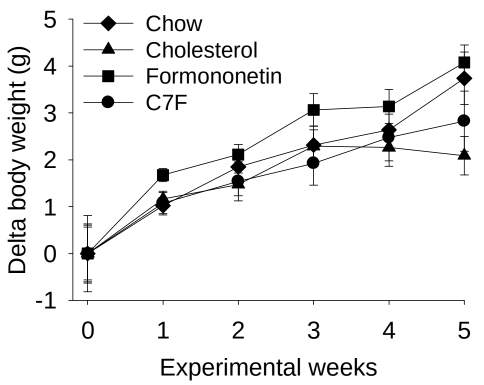

Supplementary Figure 4: Total feed intake in C57BL/6 mice fed chow, cholesterol or cholesterol supplemented formononetin or 2-heptyl-formonetin (C7F) for five weeks (experiment 2) (n=8). Graphs shows mean ± SEM.

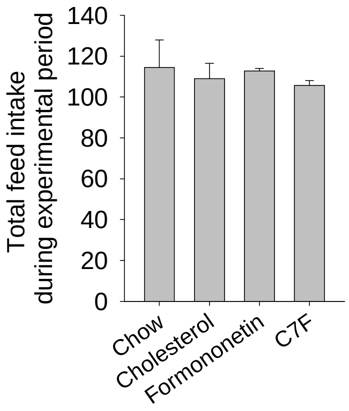

Supplement: Supplementary file 1 — Supplementary Table 1: Composition of the diets. Supplementary Table 2: A list of functional gene groupings included in the gene expression array analysis of phase I and II enzymes on C57BL/6 mice fed cholesterol supplemented formononetin or 2-heptyl formononetin (CF7) for three weeks (experiment 1). Supplementary Table 3: Primer sequences. Supplementary Figure 1: Clustering of gut microbiota from C57BL/6 mice fed formononetin or 2-heptyl formononetin (C7F) (experiment 1) (n=23-25). Three-dimensional principal component analysis (3D-PCA) based on denaturation gradient gel electrophoresis data. Black circle: cholesterol, red square: formononetin, green square: C7F. The clustering was significant on the Y-axis and the Z-axis. On the Y-axis C7F fed mice clustered significantly differently from mice fed cholesterol and the formononetin. Also the formononetin fed mice clustered significantly differently from mice fed cholesterol. On the Z-axis the C7F fed mice clustered borderline differently from mice fed cholesterol. Supplementary Figure 2: Gene expression of phase I and II metabolites (experiment 1). Scatter plot of liver gene expression (n=23-25). Gene expression in A mice fed chow compared to formononetin, B chow compared to C7F and C formononetin compared to C7F. Supplementary Figure 3: Body weight of C57BL/6 mice fed chow, cholesterol or cholesterol supplemented formononetin or 2-heptyl-formonetin (C7F) for five weeks (experiment 2) (n=8). Graph shows mean ± SEM. Supplementary Figure 4: Total feed intake in C57BL/6 mice fed chow, cholesterol or cholesterol supplemented formononetin or 2-heptyl-formonetin (C7F) for five weeks (experiment 2) (n=8). Graphs shows mean ± SEM. [file 926942.f1.pdf]
